# Draft genome sequence of *Faecalibacterium prausnitzii* FP-BI isolated from bovine feces

**DOI:** 10.1128/mra.00611-26

**Published:** 2026-08-10

**Authors:** Kapil Narayan, Nagaraju Indugu, Krishna Challa, Terry Webb, Dipti Pitta

**Affiliations:** 1 Department of Clinical Studies, School of Veterinary Medicine, University of Pennsylvania, New Bolton Center 6572, Kennett Square, Pennsylvania, USA; Wellesley College, Wellesley, Massachusetts, USA

**Keywords:** dairy calves, gut microbiome, early life

## Abstract

*Faecalibacterium prausnitzii* was isolated from Holstein calf feces. Species identity was confirmed by partial 16S rRNA gene sequencing and whole-genome sequencing. The draft genome consisted of 48 contigs totaling 3.01 Mb, with an N50 of 138,331 bp and a GC content of 56.53%.

## ANNOUNCEMENT

The early-life colonizers such as *Bacteroides*, *Faecalibacterium*, *Bifidobacterium*, and *Clostridium* in calves are pivotal to their growth and development (1). Among these, *Faecalibacterium* has been associated with improved growth and reduction in calf scours (2, 3). To date, no *Faecalibacterium* species has been genomically characterized from bovine hosts.

In this study, *F. prausnitzii* was isolated from dairy calves (Animal Ethics, IUCAC # 807522) under stringent anaerobic conditions (4) using a modified YCFA (yeast extract, Casitone, and fatty acid-enriched) medium containing fructose (0.25%) and sucrose (0.25%), supplemented with 30% autoclaved rumen fluid obtained from dairy cattle, as previously described (5). Approximately 2 g of pooled fresh feces collected from <3-week-old Holstein calves was suspended in 10 mL anaerobic diluent (prepared under CO_22_, dispensed into Hungate tubes, and sealed) to form a slurry and then centrifuged (5000 rpm, 1 min). The supernatant (0.1 mL) was serially diluted up to 10^−10^, inoculated into YCFA agar tubes, and subsequently rolled and incubated at 37°C for 24 h. Individual colonies from 10^−7^ to 10^−9^ agar dilution tubes were picked, transferred to liquid broth, and cultured. This procedure was repeated until purified bacterial cultures were obtained. Upon confirmation by microscopy for uniform morphology, the pure culture was used for genomic DNA extraction using the RBB + C method and PCR amplified for the V1–V2 region of the 16S rRNA gene using primers F27 (5′-AGAGTTTGATCCTGGCTCAG-3′) and R338 (5′-TGCTGCCTCCCGTAGGAGT-3′), as described by Pitta et al. (6). Sanger sequencing of the 16S rRNA gene showed 99.13% sequence identity to *F. prausnitzii* strain NR_028961.1. Furthermore, the genomic DNA was subjected to whole-genome sequencing using an Illumina HiSeq platform (2 × 150 bp). A total of 7,558,889 high-quality reads were assembled *de novo* with default parameters using SPAdes v3.15.5 (7) and polished with Pilon v1.24 (8). Assembly statistics were generated with QUAST v5.3.0 (9); genome quality was assessed using CheckM v1.2.3 (10); taxonomic classification was performed with GTDB-Tk (11); annotation was completed using Bakta v1.12 (12); and antimicrobial resistance genes were identified using AMRFinderPlus v4.0.13 (13).

The draft genome comprised 48 contigs (3.01 Mb; N50, 138,331 bp; GC content, 56.53%) with 100% completeness, no detectable contamination, and ~700× coverage. GTDB-Tk identified the isolate as *F. prausnitzii* with 99.99% average nucleotide identity to the reference genome GCF_003324185.1. Annotation identified 2,751 coding sequences, including 1 CRISPR array, 3 rRNA genes, 52 tRNAs, and 35 ncRNA regions, and no antimicrobial resistance genes were identified.

## Data Availability

The whole-genome shotgun project has been deposited in GenBank under accession JBWJAH000000000, with BioProject PRJNA1439328. The raw sequencing reads are available in the Sequence Read Archive (SRA) under accession SRR37735404, and the Sanger-derived 16S rRNA gene sequence has been deposited in GenBank under accession PZ688188.
